# High survivorship of highly cross-linked polyethylene in revision Total hip Arthroplasty: a minimum 10-year follow-up study

**DOI:** 10.1186/s42836-019-0017-1

**Published:** 2019-12-17

**Authors:** Seung-Jae Lim, Ingwon Yeo, Chan-Woo Park, Kyung-Jae Lee, Byung-Woo Min, Youn-Soo Park

**Affiliations:** 1 Department of Orthopedic Surgery, Samsung Medical Center, Sungkyunkwan University School of Medicine, 81 Irwon-ro, Gangnam-gu, Seoul, 06351 South Korea; 2 Department of Orthopaedic Surgery, Dongsan Medical Center, Keimyung University School of Medicine, Daegu, South Korea

**Keywords:** Revision total hip arthroplasty, Highly cross-linked polyethylene liner, Outcome

## Abstract

**Purpose:**

Highly cross-linked polyethylene has been introduced to decrease osteolysis secondary to polyethylene wear debris generation. However, few long-term data on revision total hip arthroplasty (THA) using highly cross-linked polyethylene liners are available. The objective of this study was to determine long-term outcomes of a highly cross-linked polyethylene liner in revision THA.

**Materials & methods:**

We evaluated 63 revision THAs performed in 63 patients using a highly cross-linked polyethylene liner between April 2000 and February 2005. Of these, nine died and four were lost to follow-up. Thus, the final study cohort consisted of 50 patients (50 hips), including 26 males and 24 females with a mean age of 53 years (range, 27–75 years). Mean follow-up was 11 years (range, 10–14 years).

**Results:**

The mean Harris hip score improved from 44 points preoperatively to 85 points at the final follow-up. No radiographic evidence of osteolysis was found in any hip. The mean rate of polyethylene liner wear was 0.029 mm/year (range, 0.003 to 0.098 mm/year). A total of 5 hips (10%) required re-revision arthroplasty, including one cup loosening, one recurrent dislocation, and three deep infections. Kaplan-Meier survivorship with an end point of re-revision for any reason was 91.1% and for aseptic cup loosening was 97.9% at 11 years.

**Conclusion:**

At a minimum of 10 years, the highly cross-linked polyethylene liners showed excellent clinical performance and implant survivorship, and were not associated with osteolysis in our patients with revision THAs.

## Introduction

The clinical outcomes of revision total hip arthroplasty are often inferior to those obtained with primary total hip arthroplasty (THA) [1]. Due to increasing numbers of patients undergoing THA and revision THA, improving the longevity of implant is highly desirable. Aseptic loosening has been known as the most frequent cause of revision after THA, even after revision THA [2, 3]. Aseptic loosening is associated with polyethylene wear debris that can stimulate an adverse local host response, resulting in bone resorption and aseptic loosening of the prosthesis [4].

Highly cross-linked polyethylene (HXLPE) materials are developed specifically to reduce polyethylene wear and subsequent wear debris-induced osteolysis. Previous studies have clearly shown that HXLPE liners in primary THA have superior *in vitro* [5, 6] wear rates and mid-term to long-term *in vivo* [7–9] wear rates. Recently, Feng *et al*. [10] have shown excellent clinical results of cementless THAs using HXLPE liner with a mean 12.9-year postoperative follow-up (range, 7–18 years), demonstrating the potential benefits of this new material in providing longevity even to revision settings. The potentially important difference at revision surgery may be the increased possibility of third-body wear. Hip simulator wear tests have shown the superior wear behavior of HXLPE compared with non-HXLPE when aluminum oxide particles (for the simulation of ceramic particles) or bone cement, containing barium sulfate, were added to the wear test as third-body wear particles while articulating against 28-mm cobalt-chromium (CoCr) alloy femoral heads [11]. The improved wear performance of HXLPE over non-HXLPE suggests that HXLPE may perform well in a third-body abrasive wear environment [12]. However, the long-term clinical results of HXLPE liner in revision THA are not well known because it is difficult to study a large cohort of patients with substantial clinically-rich information. We have been performing revision THAs routinely using electron beam-irradiated and melted HXLPE (Longevity; Zimmer, Warsaw, IN, USA) as an articulating liner with titanium acetabular cup to assure good performance even in revision settings.

Therefore, we performed this study with 2 objectives. The first objective was to determine the clinical and radiographic results of patients who underwent revision THA using HXLPE liner at a minimum follow up of 10 years. The second objective was to determine the linear and volumetric wear rate of HXLPE in these hips.

## Materials and methods

The study protocol was approved by the Institutional Review Board. We reviewed the records of a consecutive series of patients with revision THA who had their hips replaced using HXLPE liners. We retrospectively evaluated 63 revision THAs performed in 63 patients using Longevity HXLPE liners between April 2000 and February 2005. Of these patients, 9 died and 4 were lost to follow-up before the end of the 10-year evaluation. Thus, the final study cohort consisted of 50 patients (50 hips), including 26 males and 24 females with a mean age of 53 years (range, 27–75 years) at time of revision THA. Their mean body mass index was 23 kg/m^2^ (range, 17–35 kg/m^2^). The study cohort of HXLPE liners was primarily revised for aseptic loosening (37 of 50 [74%]), infected hip arthroplasty (7 of 50 [14%]), and polyethylene wear and osteolysis (6 of 50 [12%]). Of the total 50 hips used for this study, 32 (64%) were performed for isolated cup revision, 12 (24%) for simultaneous cup and stem revision, 5 (10%) for liner cementing, and 1 (2%) for isolated liner change. All infection-related causes were treated with 2-stage revision arthroplasty. The mean duration of follow-up was 11 years (range, 10–14 years). A summary of the demographic data is provided in Table 1.

**Table 1:** Demographic data

Number of patients (hips)	50 (50)
Age at revision surgery (years)	53 (27–75)
Gender (Male:Female)	26: 24
Body-mass index (kg/m^2^)	23 (17–35)
Cause of revision surgery (%)	
Aseptic loosening	37 (74)
Infection	7 (14)
Polyethylene wear and osteolysis	6 (12)
Duration of follow-up (years)	11 (10–14)
Values are presented as mean (range).	

### Prosthesis

All surgeries were performed at a single institution by a single experienced surgeon. The Longevity HXLPE liner was used in all hips. Uncemented cups and uncemented stems were used in all hips. Cementless titanium acetabular cups (Trilogy Acetabular Hip System; Zimmer) were press-fitted (1–2 mm) with 1 or 2 screws used for supplemental fixation. The femoral head size was selected to maintain a polyethylene thickness of approximately 6 mm to avoid problems associated with thin polyethylene, particularly at the rim of the acetabulum. Average thickness of polyethylene was 8.9 mm (range, 6.1–14.3 mm). It was calculated using Zimmer’s Longevity product brochure [13]. A cobalt-chromium femoral head was used in 36 hips (28 mm head in 33 hips and 22 mm in 3 hips). A 3rd-generation alumina ceramic femoral head was used in 14 hips (28 mm in 14 hips). Although the decision for use of a cobalt-chromium or ceramic head was at the operating surgeon’s discretion, a ceramic head was not used in situations of damaged stem tapers. As mentioned above, femoral component was replaced simultaneously for 12 hips.

### Evaluation

Each patient was clinically and radiographically assessed 4 weeks, 3 months, 6 months, 12 months after surgery, and annually thereafter. Clinical outcomes were assessed using a physical examination and Harris hip score (HHS) [14] at each follow-up interval. Standard radiographs included anteroposterior (AP) and translateral view of the hip. Radiographs taken 4 weeks after the index operation served as the baseline for all subsequent comparisons. These radiographs were analyzed for wear, radiolucent lines, osteolysis, or a change in the position of the component over time. Wear of HXLPE was measured using Polywear technique [15]. Radiographs were evaluated for loosening (defined as a change in component position of greater than 5 mm or a circumferential radiolucent line of 2 mm or greater) [16]. Acetabulum and femur were evaluated for evidence of osteolysis (defined as a nonlinear radiolucency greater than 5 mm of the bone adjacent to the prosthesis). Findings were recorded for 3 zones of the acetabulum described by DeLee and Charnley [17] and 7 zones of the femur described by Gruen *et al*. [18]. The position of the acetabular component was determined according to the method of Woo and Morrey [19]. Abduction was measured on the AP pelvic radiograph as the angle formed by lines drawn tangential to the acetabular component and tangential to the horizontal line joining the teardrop. Anteversion was measured on the true lateral radiograph as the angle formed by a line drawn tangential to the face of the acetabular component and a line drawn perpendicular to the horizontal plane. Femoral component stability and osseointegration were assessed using method described by Engh *et al*. [20].

Polyethylene wear was determined from annual radiographs using computer-assisted method with PolyWare software (Draftware Developers, Vevay, IN) [21]. This software can read tagged image file format (TIFF) files digitized from radiographs. It can identify the center of the femoral head and acetabular shell from the detected edges automatically. In addition, it can calculate the linear wear by distance between two points. All radiographs were measured by the same observer. To reduce disproportionate effects of embedding and creep, the initial radiograph used was the one taken 6 months after surgery [22]. Every X-ray picture was measured 5 times. After excluding the highest and lowest value, the mean value of the remaining 3 results was calculated. Intra-observer reliability was determined using PRO Mixed procedure of SAS program (Information Technology Services; Austin, TX, USA). The intra-observer error was within 0.025 mm. The end point of this study was the first re-revision THA (for aseptic or septic reasons). Re-revision was defined as any operation in which a previously implanted component was replaced.

### Statistical analysis

All statistical analyses were performed using PASW Statistics version 23.0 (IBM Corp., Armonk, NY). Student’s *t*-test was used to compare preoperative and postoperative HHS. The polyethylene wear data of the CoCr and ceramic femoral head groups were compared with use of the Mann-Whitney U test. Kaplan–Meier survivorship analysis was performed for all hips, with re-revision of either component as an end point. Statistical significance was considered when *P* value was less than 0.05.

## Results

### Clinical outcomes

The mean HHS significantly (*P* < 0.001) improved from 44 points (range, 24–89) preoperatively to 85 points (range, 52–100) at the final follow-up. No patient required any kind of walking support at the time of the latest follow-up.

### Radiographic results

**Fig. 1:**
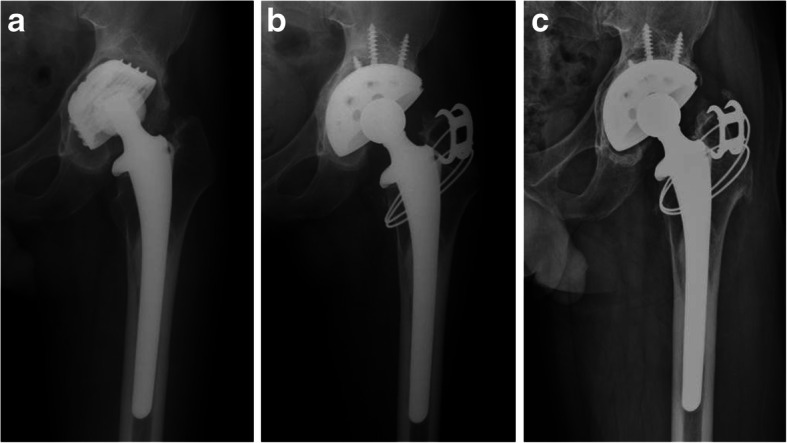
(**a**) Preoperative radiograph showing aseptic loosening of acetabular cup; (**b**) Postoperative radiograph of cementless total hip arthroplasty performed with Longevity higly cross-linked polyethylene liner and 28-mm metal head; (**c**) Twelve-year follow-up radiograph showing well-fixed prostheses without osteolysis. The polyethylene liner wear rate was 0.014 mm/year

The average abduction angle and anteversion of the acetabular component were 41.2° (range, 43° to 56°) and 14.7° (range, 8° to 23°), respectively. All acetabular components were fixed by bony ingrowth except 1 hip, for which the cup was replaced due to aseptic loosening (Fig. 1). Three hips (6.0%) had radiolucent lines around the acetabular component without the evidence of component loosening. At the femoral side, the radiolucent lines were found in 5 hips (10.0%) in zones 1 and 7, however, they did not progress during the observation period. No hips had > 5 mm of subsidence of the femoral stem.

### Condition of wear

The mean rate of polyethylene liner wear was 0.029 mm/year (range, 0.003 to 0.098 mm/year). With the numbers available, there was no significant difference between the CoCr and ceramic femoral head groups with regard to the rate of polyethylene liner wear (*P* = 0.751). At the final follow-up, no hip was an outlier based on the threshold of osteolysis at 0.10 mm per year [23, 24] because all liners had wear rates below this level.

### Re-revision and survivorship

**Fig. 2:**
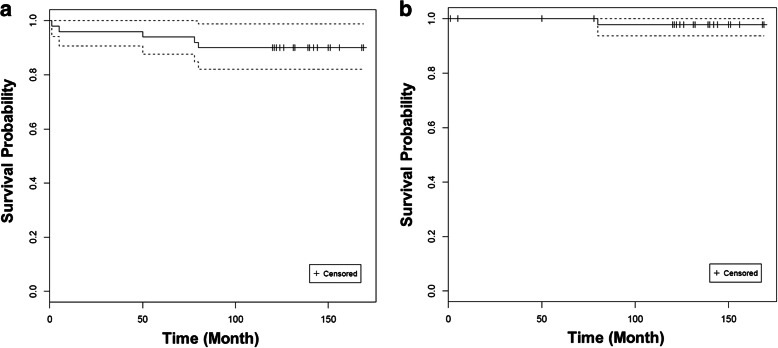
Kaplan-Meier survival analysis of revision THAs using HXLPE liner. (**a**) Survival with re-revision for any reason as the end point; (**b**) Survival with re-revision for aseptic loosening as the end point. Dotted lines indicating 95% confidence interval

During the follow-up period, 5 (10%) hips required re-revision arthroplasty. One hip required re-revision surgery due to aseptic acetabular cup loosening at 6.5 years postoperatively. One required re-revision with a constrained liner due to recurrent dislocations. Three sustained deep infections required additional surgical procedures. However, none of the liners was revised due to polyethylene wear or mechanical failure of the polyethylene. The characteristics of patients who were re-revised are summarized in Table 2. The Kaplan-Meier survivorship with an end point of re-revision for any reason was 91.1% (95% confidence interval [CI], 86.7 to 100%) at 11 years. For aseptic cup loosening, the Kaplan-Meier survivorship was 97.9% (Fig. 2).

**Table 2:** Characteristics of patients with re-revision for reasons other than periprosthetic fracture

Patient no.	Sex	Age at revision	Reason for revision	Method of revision	Acetabular cup at revision (size)	Acetabular liner at revision (thickness)	Femoral head at revision (size)	Acetabular cup inclination at revision (°)	Acetabular cup abduction at revision (°)	Months from revision to re-revision	Reason for re-revison	Method of re-revision
1	M	43	Aseptic loosening	Cup revision	Trilogy (66 mm)	Longevity (13.3 mm)	Metal (28 mm)	38.3	9.3	80	Aseptic loosening	Cup revision
2	F	65	Aseptic loosening	Cup & stem revision	Trilogy (48 mm)	Longevity (6.2 mm)	Ceramic (28 mm)	44.0	12.5	6	Recurrent dislocation	Cup revision
3	F	62	Aseptic loosening	Cup revision	Trilogy (48 mm)	Longevity (6.2 mm)	Metal (28 mm)	38.0	10.5	78	Infection	2 stage cup & stem re-revision
4	M	75	Infection	Cup & stem revision	Trilogy (64 mm)	Longevity (12.3 mm)	Ceramic (28 mm)	36.4	8.5	1	Infection	Resection arthroplasty
5	M	56	Aseptic loosening	Cup revision	Trilogy (56 mm)	Longevity (8.3 mm)	Metal (28 mm)	49.1	13.0	50	Infection	2 stage cup re-revision

### Complications

There was no evidence of intraoperative periprosthetic fractures or intraoperative acetabular loosening. No case of sciatic nerve palsy, symptomatic venous thromboembolism, or surgery-related mortality was observed. However, during the follow-up period, there were 2 cases of postoperative periprosthetic fractures (1 acetabular fracture and 1 femoral fracture) associated with high-energy trauma. These fractures healed after open reduction and internal fixation.

## Discussion

Aseptic loosening and osteolysis are the most common causes of revision surgery following THA after a long term [2, 3]. One approach to reduce aseptic loosening and osteolysis was to modify the material properties of bearing surfaces [25, 26]. HXLPE liners have been developed with improved resistance to wear and fewer wear particles generated *in vitro* [5, 6]. Several authors have studied the durability of HXLPE bearings in hip arthroplasty and reported favorable radiographic and clinical outcomes of HXLPE bearings in primary settings [7–10]. Our previous study has also found that HXLPE bearings used in young patients with osteonecrosis of femoral head are less likely to be replaced for extensive wear and osteolysis than conventional polyethylene bearings in revision settings [27]. The potentially important difference at revision surgery may be the increased possibility of third-body wear. Hip simulator wear tests have shown the superior wear behavior of HXLPE compared with non-HXLPE in a third-body abrasive wear environment [11, 12]. However, the long-term clinical results of HXLE liner in revision THA are not well known.

The first objective of this study was to determine the clinical and radiographic outcomes of cementless revision THA using current techniques and HXLPE bearings with a minimum follow-up of 10 years. In our study, Kaplan-Meier survivorship with an end point of re-revision for any reason was 91.1% (95% CI: 86.7 to 100%) at 11 years. We found these types of bearings had good mid- to long-term follow-up results. This has not been previously reported in revision settings. Implant survivorship in our study is comparable to that of primary THA. A recent large population-based study has shown that cumulative incidence for any revision is 5.3% for metal on PE in primary THA after follow-up of 8.3 years [28]. Monti *et al*. [29] recently reported that the age was the only patient-associated risk factor for re-revision THA. According to that study, for every ten-year increase in patient’s age, the hazard for re-revision is significantly (*P* = 0.004) decreased by a factor of 0.72 (95% CI, 0.58 to 0.90). The mean age at revision surgery was 53 years in this study. Considering the relatively younger age of these patients at revision surgery, our implant survival rate was excellent. HHS, the most commonly used scoring method, significantly improved postoperatively, with a mean of 85 (80–90 constitutes a “good outcome”) at the final follow-up.

The second purpose of this study was to determine the linear and volumetric wear rate of HXLPE in revision THA using HXLPE bearings at a minimum follow-up of 10 years. Our results revealed that the mean rate of polyethylene liner wear was 0.029 mm/year (range, 0.003 to 0.098 mm/year), comparable to that of HXLPE in primary THA. Reynolds *et al*. [30] have conducted a comparison study for all current wear rate of HXLPE in primary THA and found that the range is from 0.002 to 0.15 mm/yr. We used AP hip radiographs to measure polyethylene wear rate. The beam is centered over the hip, which may be more orthogonal to the vector of penetration. Nimrod *et al*. [31] found a significant difference in measurements of linear penetration when comparing AP pelvis to hip radiographs. Lower rates are found to be recorded when AP pelvis is used. Therefore, our result has less chance to underestimate the amount of penetration. The excellent clinical and radiographic results of HXLPE liners suggested that free radicals and oxidation do not appear to accelerate annealed HXLPE liner wear rate during this time period. Through the current study on HXLPE liners with a minimum follow-up of 10 years, we found that these types of bearings had reliable mechanical properties even in mid- to long-term situations. This has not been previously reported in revision settings.

There are several possible reasons for the success of our revision THAs other than HXLPE liners itself. It has been suggested that reduced strength and toughness of HXLPE may play an important role in the fracture of liner rims and the locking mechanisms [32, 33]. These altered mechanical properties of HXLPE can be adversely affected by acetabular inclination, polyethylene thinness, or both. The mean inclination angle of acetabular cup and mean thickness of HXLPE liners in this study were 41.2 and 8.9 mm, respectively. These inclination angle and thickness of liner were in acceptable ranges according to recent studies, which might have prevented unnecessary re-revision surgeries. We performed all revision THA using uncemented stems. Fixation type and the risk of re-revision have been studied extensively. Multiple contemporary studies have reported highly successful outcomes of revision THAs using uncemented stems [34, 35]. In addition, the operator had been performing more than 50 revision THAs annually in our institution. Some authors have also suggested that increased experience of surgeon can improve the outcome of technically challenging hip arthroplasty. Khatod *et al*. [29] have reported that the hazard of re-revision THA is significantly (*P* = 0.049) decreased by a factor of 0.93 (95% CI: 0.86 to 0.99) for every 5-unit increase in the number of revision arthroplasties performed by the surgeon.

Many authors have also paid attention to materials of femoral head and searched for ideal pairings of acetabular liner and femoral head in hip arthroplasty. For an existing taper during revision surgery, it is generally not recommended to use a ceramic head because undetected damage in the taper may increase the risk of ceramic fracture [36–38]. In addition, a component mismatch may lead to accelerated wear and earlier revision [39]. In our patients, 36 hips received CoCr femoral head and HXLPE liner while 14 hips received ceramic femoral head and HXLPE liner. A recent multicenter randomized controlled trial on polyethylene wear rate showed that no significant (*P* = 0.153) difference was noted between CoCr and ceramic head when articulating with HXLPE [40]. Based on this trial, it appears that using a HXLPE acetabular liner is more important in reducing THA component wear than the choice of femoral head bearing with mid-term follow-up time. Therefore, we did not pay attention to the difference between the two materials of femoral head.

This study had several limitations, including its retrospective nature. In addition, this was not a comparative study. Therefore, conclusions cannot be drawn about the outcomes of HXLPE liner compared to ceramic liner. Moreover, we had a relatively small number of patients compared to studies on primary THA with a large number of patients. However, only a small cohort of patients was available for observation due to the unique usage of this bearing system for revision THA. Furthermore, no long-term data are available at this point. In addition, only one experienced adult reconstruction surgeon from a single high-volume center performed all these revisions. Thus, the outcomes may not be generalized to other centers with lower volumes. The technique used to evaluate our patients’ polyethylene wear is also a possible limitation because alternative methods can be used to measure wear [41].

## Conclusions

At a minimum of 10 years follow-up, our results revealed that the use of HXLPE liner in revision settings was promising even in a cohort of our patients with a mean age of 53 years. Patients’ clinical scores were improved. From a radiological point of view, only one aseptic acetabular loosening took place after a minimum of 10 years after implantation. Based on the encouraging outcomes in this challenging revision THA, we believe that cementless THA using HXLPE liner is a promising procedure. Due to the intermediate follow-up time and the relatively small study group in a highly specialized institution, these results may not be extrapolated to the general population. Further evaluation for this patient group after a longer follow-up time is needed in the future.

## Data Availability

The datasets used and/or analysed during the current study are available from the corresponding author on reasonable request.

## Abbreviations

THA: total hip arthroplasty
HXLPE: highly cross-linked polyethylene
CoCr: cobalt-chromium
HHS: Harris hip score
AP: anteroposterior
